# American Dental Association

**Published:** 1886-02

**Authors:** 


### AMERICAN DENTAL ASSOCIATION.

### TWENTY-FIFTH ANNUAL MEETING, HELD AT MINNEAPOLIS, MINN., AUGUST 4, 5, 6, AND 7, 1885.

REPORTED FOR THE INDEPENDENT PRACTITIONER BY "MRS. M. W. J."

(Continued from page 31.)

### THURSDAY AFTERNOON SESSION.

Dr. Wm. H. Atkinson read a paper entitled "Pyorrhœa and Sponge-grafting," of which the following is an imperfect abstract:

He said that correct diagnosis was the first prerequisite; that there was no real pyorrhœa without loss of attachment of the *ligamentum dentium;* a flow of pus was always an evidence of nature's failure to form new tissues. Pyorrhœa was cured by destroying the microbes which prevent granulation; that an imperfect elimination of urea was an antecedent of pyorrhœa; that traumatic pyorrhœa was the resultant of the presence of foreign bodies in the alveolus, as particles of bone, splinters of wood, etc. These must be removed and the pockets sterilized. Calcaerous deposits were not always present, but if they were they must be removed and their re-formation prevented. Many parisiticides which would destroy microbes would form eschar tissue, changing pabulum into cooked flesh. Nitrate of silver would afford protection by supplying an outer wall. Non-sterilized pockets might discharge pus for an indefinite length of time. There is lack of knowledge of molecular changes. His object in writing was to state the results of his experience. He would prescribe tonics, as sulphate of quinine. For local treatment :—

1st. When there is slight recession, and no discharge, use elixir vitriol.

2d. When the loss of attachment is greater, use Aqua Regia, 1 to 3.

3d. Where the loss is very great, the gums dark, with discharge of pus and bloody serum, use the carbolized potash paste, known as Robinson's Remedy, not allowing it to overflow on the lips or cheek.

4th. When the roots are largely exposed, and there is no opportunity for covering in the pockets to induce new growth, use the sponge-graft. This is a recent discovery, of which little is generally understood, but than which there is nothing of greater value. It grew out of the misfortunes of traumatic lesions, having been first used by obstetricians to supply gaps caused by violent labor. The sponge is wrung out of bi-chloride of mercury. Tents must be removed and changed, but the sponge remains permanently. We have advanced by stages from the actual cautery, boiling oil, red-hot iron, for the arrest of hemorrhage, followed by the irrigation and drainage of wounds, to the transplanted skin and epithelial tissue. This suggested some material to take the place of lost substance. Many forms of animal sutures and flesh tents were used, but these were generally extruded and cut off. It is not true, however, that chicken flesh, etc., was ever used to fill gaps. Sterilization or Listerism was a great step in advance. Then came the sponge graft. A gaping wound, filled with sterilized sponge, and covered over with plaster to support and prevent exudation of blood and serum, will heal by first intention if left undisturbed. All germs must be destroyed. There is a large list of germicides, but bi-chloride of mercury is the best. Proper discrimination must be used in judging of the favorable or unfavorable progress of the healing process.

*Dr. J. S. Marshall*—Wished to ask Dr. Atkinson how to protect the sponge-graft after it was placed in position in the mouth for the cure of loss of substance by pyorrhœa.

*Dr. Atkinson*—After thorough removal of all deposits, tie loose teeth firmly, having the occlusion perfect; then take an impression and make a cast. Wherever the tissue is missing build it up with wax, rather higher than the new growth is wanted. Make a cast and strike up a plate, making a tight pocket which will fit the teeth closely and prevent anything from escaping; put the sterilized sponge inside of this pocket. In larger places, as from removal of tumors or morbid growths, cover in with flaps of skin, leaving the clot of exudation. It does not form scar-tissue. It is reproduced tissue. Do not undertake to buy sterilized sponge. Buy surgeon's fine sponge, see that it is free from all foreign elements, and sterilize by dipping in a solution of one grain of bi-chloride of mercury to one ounce of distilled water at 130° Fahrenheit. At or above 133°

the sponge will shrivel, and 160° will cook it, so that it is not fit to use, as the living material that is designed to be absorbed is killed. If the sponge is not thoroughly sterilized the wound will not heal by first intention. Do not remove the sponge for any cause whatever. If pus should ooze from one corner, or the sponge get black or green, and hard, where pabulum has not penetrated, wipe off the serum, and with fine pointed scissors cut off the discolored portions till red pabulum (arterial blood) is reached. Sterilize the sponge again, fitting a little bit into the vacant space, not disturbing the adhering portions. Use simple common sense. Any man that can fill a tooth can be a good surgeon.

*Dr. Marshall*—Said that in his previous attempts the sponge would get displaced from the pyorrhœa pockets, and he had almost concluded that the graft was useless. With Dr. Atkinson's method, as just described, he had no doubt of success in the future. He had tried Dr. Brigg's method of preparing the sponge by placing it in dilute hydrochloric acid, treating with ether and iodoform, but the sponge dissolved and was good for nothing. In certain forms of perforation, or cleft palate, he thought the sponge-graft might be practical and valuable.

*Dr. Friedrichs*—Inquired if it would not be necessary to denude the surface.

*Dr. Marshall*—Of course. That has to be done in every case.

*Dr. Abbott*—Said that he was pleased to show the method of using his Spray instrument for applying remedies in the mouth, with which they could be thrown wherever desired, as back of the soft palate, up into the posterior nares, etc. In abscess of the antrum, remedies could be applied directly to every point of the cavity; in case of hypertrophy, there were more or less layers in the mucous membrane discharging pus. These should all be cleansed; the syringe could only wash off the surface, while this instrument would carry it in every direction at the same time, filling the cavity full. Dr. Farrar had invented a syringe with similar points, but this was a more simple and more certain way of applying remedies, as the spray had intense force. In using remedies that were liable to crystallize, as the boracic acid of listerine, water must be drawn through after each application, for cleansing. The instrument is not confined to the use of listerine. Any liquid can be used. It has sixty pounds' pressure, and is invaluable for the treatment of diseases of the antrum. It is valuable for blowing chips from the cavity, as it gives a continuous stream of air ; it is also excellent for local anæsthetizing, the tooth being rendered insensible to pain in a moment's time.

*Dr. Taft*—Suggested its use with the warm air blow-pipe.

*Dr. Abbott*—Replied that an alcohol lamp or gas jet could be introduced underneath for keeping the air warm, heating compressed air not increasing its force, even if raised to 120° or 140°.

*Dr. Taft*—Said that 200° would increase its tensity, and it would be as well to advise caution, and not to go above a certain temperature.

*Dr. Abbott*—Said there would be no risk, as it would never be necessary to go above the heat of the body.

Adjourned to 8 P. M.

THURSDAY EVENING SESSION.

Dr. Kulp asked for information concerning the section of Dental and Oral Surgery of the International Medical Congress.

Dr. Allport, chairman of the committee, replied.

After some discussion the subject was postponed.

A letter was read from Dr. Clifton, of Waco, Texas, thanking the members of the Association for kind attentions and sympathy at the time of the accident to his little son during the Saratoga meeting.

On motion, Section VI was passed.

Section I, Prosthetic Dentistry, Metallurgy and Chemistry, was called.

Dr. Wm. H. Trueman presented a paper on "A New Method of Flasking Vulcanite Rubber Work," a method which offers advantages in cleanliness, saving of time, and safety of teeth, especially in difficult partial cases. By the original English method the work was invested in steatine or soap-stone. Dr. J. Speyer, of Philadelphia, has modified the method, using the patented "Surface Cohesion Forms," a prepared tin foil, similar to that used for stipple-work, or for securing a clean, bright palatal surface. The forms are embossed with geometrical figures, which leaves a network of continuous channels, instead of a central suction cavity. This gives firmer adhesion, causes less irritation to the mucus surface, and allows a much smaller plate; even if the plate tilts the suction is not entirely lost. The portion of the model to be covered with the form is thickly painted with a cement made of vulcanizable rubber dissolved in chloroform to the thickness of a heavy syrup, the foil being pressed down till all the indentations are filled with the cement, which prevents crushing out of shape in packing. Rubber softened in hot water is used in mounting the teeth, instead of wax or gutta-percha base plates, small bits of softened rubber and the cement being packed in around the teeth and pins. The rubber is built up to the proper size and shape, and finished as wax is generally used. It is then invested in plaster, without allowance for opening the flask, as there is no wax to be removed, and the cover screwed down before the plaster sets, when it is ready for the vulcanizer. While time and labor are saved, and cleanliness insured, the method has the great disadvantage of not allowing of trial in the mouth until finished.

The rubber dissolved in chloroform forms a cement which is valuable for many purposes, as for repairing bulbs of syringes, etc.

Dr. L. P. Haskell read a paper entitled "What to Do and How to Do it." He said that many dentists did not appear to apppreciate the vast difference in mouths. A patient will ask why her teeth are not as easily made satisfactory as those of Mrs. Blank. She evidently thinks the difference lies in the dentist, but it is more probably due to the difference in mouths. As we all know, some mouths are very easy to fit, while others require great care and skill and study, and the wearer is required to exercise great patience in learning to use the plate. When there is a firm ridge, not deep, the palatal surface not hard, the lower jaw not protruding, but striking fairly under, there will be no trouble in putting in a satisfactory plate. But the contrary may be the case in some or all points, as when there has been great absorption, with flexible ridge; and this condition we find, to a fearful extent, under vulcanized rubber plates by which thousands of mouths have been ruined; the better the adaption the worse will be the results. When the plate is loose-fitting less damage is done; where the suction is perfect, an abnormal condition prevails from undue retention of heat. It is not correct to say that the process is absorbed; it is simply that waste material is not replaced. For this reason rubber plates are doing incalculable damage; the Alveolar process is often entirely gone, and the plate rests upon the muscles and soft tissues.

Skill must be used in fitting the plate and in antagonizing the teeth. Many plates are apparently worthless, simply from faulty antagonizing, which a little skillful grinding would make quite useful and comfortable. All conditions of the mouth are found ranging between the two extremes. In preparing the mouth for artificial teeth, the extraction of the remaining teeth is a point requiring especial consideration. While it is true that it is the province of the dentist to save the natural teeth, when the time has come that the patient must wear some artificial teeth the question arises, shall it be a full or a partial set, when the former involves the extraction of some remaining teeth? A safe rule to follow is to remove whatever will interfere with the comfort and utility of the plate, throwing aside all sentiment regarding sound teeth, if they are in the way of making the plate completely comfortable and useful. As to the material for the plate, rubber is universally used the world over, because it is cheap and easily made. Any young man can learn to make a rubber plate in a few weeks; in a few weeks more he learns to insert amalgam fillings in simple cavities, and this constitutes the whole stock-in-trade of many who call themselves dentists. Metals, being good conductors of heat, are preferable in the mouth. When undue absorption takes place under a metal plate, as is sometimes the case, it will be found that there has been undue pressure in front, but this is exceptional. Whenever it is possible, a patient should be induced to have a metal plate. You may say that you have no demand for metal plates. You must create the demand. Ask the average patient what kind of a plate he wants, and, as a rule, he will answer, rubber. Ask him why he wants rubber, and you will find that he seldom has any better reason than because all his friends wear it. Explain to him that it is not the best, and why it is not the best, though the cheapest, and he will probably understand that the cheapest is not the best in teeth, any more than in clothing, though it is true that many who want only the very best that money can buy in clothing will go shopping to find the cheapest in dentistry.

For taking impressions, various substitutes for plaster are being used, as modeling compound, wax, etc., but for a perfectly correct impression, plaster is the only material that is absolutely reliable, and everything depends upon a correct impression. I use no air-chambers, but swage the plate up to the palate in every case. I recently saw a number of old patients who were wearing plates that I put in twenty or twenty-five years ago. In every case they were swaged up to the palate, and the alveolar process was as solid and the mouth as healthy as on the day they were put in. But of late years we find many mouths in which the process is entirely gone, and only a flexible tissue left. In such cases I raise the cast slightly in the center, with a thin film of wax, scraping out a little when necessary to make the plate set snug in the soft parts. There is but one metal that possesses all the requisites for dies, and that is genuine Babbitt metal. It has the necessary hardness, smoothness, and toughness, but you must be sure that it is Babbitt metal. If it is offered for twenty-five or thirty cents per pound, it is worthless. Lead has been substituted for tin, which makes it too soft. That made by S. S. White, or Justi, is good. The formula is 1 part copper, 2 parts antimony, and 8 parts tin. For the counter die one-eighth of tin must be added to the pure lead, or it will soften and adhere. You will seldom have to make a second die if your materials are right. One die will swage it to fit accurately. When the die fits the plaster cast, it will also fit the mouth.

Dr. Haskell said that he considered that Bridgework, or the system of teeth without plates, was doing incalculable injury. It was impossible that a plate permanently attached to the natural teeth should be otherwise than uncleanly. If placed under the nose when removed for repairs, it would be found absolutely disgusting. The teeth to which the gold band of the bridgework was attached with cement must inevitably loosen and disintegrate. It was impossible to prevent the secretions of the mouth from going there to stay, and the teeth of attachment would soon be girdled with decay, and loosened in the jaw. The method exhibited by Dr. Parmly Brown was the least objectionable, and might be admissible in some cases, but even that might be carried to extremes, and a long bridge would inevitably go at last. There was no necessity for it, except occasionally as a last resort, but in the majority of cases a narrow gold plate, with properly adjusted clasps, which could readily be removed and kept clean, would answer every purpose, and would do no harm and occasion no strain on the natural teeth.

*Dr. W. B. Ames*—Said there were but two methods of retaining upper plates: spiral springs, which were no longer used, and atmospheric pressure. The usual form of a large chamber over the center of the palate afforded unequal support, while the tissues soon filled the cavity. The plate should be so constructed as to equalize the bearing, atmospheric pressure being applied just where needed. Trimming the cast was not sufficient, but the plate must extend to a point where the edges will displace the soft tissues, running well up beneath the lip and cheeks, so as to lie snugly on the edge. Instead of raising the center, as described by Dr. Haskell, he would trim away where soft, leaving a groove across the portion of the plate where the tissues are lax. If this can not be tolerated because of nausea, he would use a soft rubber edge.

*Dr. Wm. H. Morgan*—Wished to notice one or two fundamental errors. He did not appear as the champion of rubber, which was a poor article for a base, causing troubles which would not occur with a gold plate. The first speaker from Chicago left the inference that the damage done by rubber was attributable to undue heat. This was a mistake. The mucus membrane does not eliminate heat; it could not get above blood-heat, unless there was inflammation; it could not eliminate enough heat to raise the temperature of the mouth above that of the body. The idea of undue heat was a wrong impression. There is always irritation and inflammation preceding abnormal heat. We must go back of that for the production of loose tissues in the front of the mouth. Again, it was said that all the natural teeth should be removed that would interfere with the artificial denture. The contrary should be the rule. The comfort and utility of the natural teeth should be considered, and the plate made to conform to them.

*Dr. W. P. Horton*—Asked Dr. Morgan to explain the production of the loose tissues.

*Dr. Morgan*—We do not know. It is true that it is worse under rubber plates, but it is not from undue heat.

*Dr. Atkinson*—The gentlemen who so unqualifiedly condemn bridgework have not seen it properly constructed, or they would speak differently. Perfect bridgework, intelligently attached, is as cleanly as anything possibly can be. Rubber first anæsthetizes the sensitive membranes, and then paralyzes the sensory nerves; it allows the blood to flow in and form little lakes, destroying the lime-salts. If we knew what we were about, we would have no difficulty with shopping-people in our offices. I want my patients to regard me as an honest man, with a conscience. I say to them: "I will do the clean thing by you, as I am an honest man; love me well enough to believe that I will do what is right." People do not want nasty things in their mouths, and rubber is nasty beyond all things.

*Dr. Haskell*—Dr. Atkinson has answered Dr. Morgan admirably. As regards bridgework I excepted the method advocated by Dr. Atkinson, as practiced by Dr. E. Parmly Brown, it being cleanly if properly inserted.

*Dr. Morgan* (to Dr. Atkinson)—Did you say there could be heat above the normal, without irritation ?

*Dr. Atkinson*—No. There is always irritation and inflammation under a rubber plate.

*Dr. C. W. Spalding*—If you will take the trouble to test the actual temperature under a rubber plate, and under a gold plate, you will not find four degrees of difference. Anyone can make the test and satisfy himself.

*Dr. T. W. Brophy*—It was well said that the tissue becomes inflamed under rubber plates. Why ? Because the vegetable substitutes not being good conductors, cold contracts the capillaries around the plate, but not beneath it. The blood rushes to the capillaries beneath and forms little lakes of blood. Then follows congestion and absorption ; not because overheated under the plate, but because the surroundings are of unequal or uneven temperature. The inequality of temperature beneath and around the plate causes the trouble.

*Dr. Atkinson*—The closing of the mucus follicles will produce mischief. If the plate is worn during sleeping hours the secretions will be changed to pus. I have seen it all over the roof of the mouth. I knew a dentist who wore celluloid. I looked in his mouth and found it all over aphthous patches, due to the paralyzing of the sensory nerves. He was cured in six months by wearing a metal plate. No plate should be worn all night.

### FRIDAY MORNING SESSION.

Dr. A. M. Dudley moved that the chair appoint a committee of seven to consider the feasibility of holding an International Dental Congress in 1887, said committee to correspond with the various dental bodies and leading men in the profession, and report at the next meeting of the Association.

The motion was carried without discussion.

The chair appointed:

Dr. Dudley, Salem, Mass., chairman.

Dr. C. N. Pierce, Philadelphia.

Dr. Frank Abbott, New York.

Dr. M. W. Foster, Baltimore.

Dr. T. W. Brophy, Chicago.

Dr. A. H. Thompson, Topeka.

Dr. Jos. Bauer, New Orleans.

Section VII reported as subjects for investigation during the ensuing year:

Normal and Abnormal Oral Fluids.

The Tongue in Health and in Disease.

The Personal Hygiene of Dentists.

On motion of Dr. Harlan, a vote of thanks was tendered the railroads, steamboats, and hotels, the press of Minneapolis and St. Paul, the Dental Societies of Minnesota and Minneapolis, and the Local Committee.

Thanks were also returned to the Trustees of the Curtiss Commercial College, where the meetings of the Association were held.

After some further announcements relative to the proposed excursions to Lake Minnetonka, the Flouring Mills, and the Yellowstone Park, Dr. E. Parmley Brown read a short paper entitled "Dentistry ; its Past, its Encouraging Present, its Brilliant Future."

He said that Dentistry, once looked upon as almost vulgar by the masses, had been hybridized with truth, impregnated with learning, illuminated by art and stimulated by poetry. That while medicine was almost a pure science, and surgery an artistic science, dentistry was a scientific art ; strictly artistic in the execution of its practice, and scientific in the preparation of the workman for his work. That dentistry was not a part of anything, but a something complete within itself ; closely allied to medicine and surgery, but more operative and more artistic than either. That dentistry, while in no sense a specialty of medicine, yet had its own specialties, as dental surgery, operative dentistry, prosthetic dentistry, anæsthetic dentistry, regulating dentistry, etc.; more than any one brain and body could master in all its details. The carcass of the past is fertilizing the luxurious growth of the present; the future it was not possible to predict. The successful dentist of the future must be an educated and polished gentleman, in addition to being master of his profession. The lady of the future would select her dentist with ten times more fastidiousness than she will her physician. The dentist must choose wisely between rusting out and wearing out, to make sure of holding out. It was violating the laws of physiology, philosophy and christianity, to labor unceasingly, and it transmits an evil influence to posterity. There is no more wisdom in overwork than in becoming entirely miserable in trying to be happy. The dentist who tries to do without sunlight will be but a sickly plant, a stunted tree. The growth of dentistry had been like the progress of the means of illumination; beginning with the ignition of a pine-knot with the spark from a flint, to the moulded tallow candle, the friction match, and illumination by gas, and finally, the dazzling light of electricity. Comparable to the latter are the microscopic investigations and the ingenious appliances of the dentistry of the present. Ideal dentistry will keep pace with all other things good for man's advancement. An appreciative public, creating a demand for it, will produce it.

The next order of business was the selection of the next place of meeting. The Executive Committee reported that they had received invitations from Louisville, Buffalo, St. Louis, Detroit, New Orleans, Deer Park, Oakland, Niagara, Newport, R. I., Cresson Springs, Pa., Lake George, Asbury Park, N. Y., and other places.

Dr. Atkinson moved that the matter be referred to the Executive Committee, with power to confer with railroads, hotels, etc., and select the place of meeting, the same to be announced through the journals.

The constitution requiring that the place be chosen by ballot, the rules were suspended and the motion carried unanimously.

The next order of business was the election of officers. Of 119 votes cast for President on the first informal ballot, Dr. W. C. Barrett, of Buffalo, having received a majority over all, the ballot was declared formal, and his election made unanimous.

Dr. L. C. Ingersoll, of Keokuk, Iowa, was elected 1*st Vice-President.*

Dr. A. T. Smith, of Minneapolis, Minn., 2*d Vice-President.*

Dr. Geo. H. Cushing, of Chicago, *Recording Secretary.*

Dr. A. W. Harlan, of Chicago, *Corresponding Secretary.*

Dr. Geo. W. Keely, of Oxford, Ohio, *Treasurer.*

Dr. A. H. Thompson offered the following resolution :

*Resolved.*—That as an expression of the appreciation of the valuable services of Dr. J. N. Crouse, President of this Association for the closing year, that he be presented with the gavel which he has so effectively wielded, as a souvenir of this successful and enjoyable meeting, and the same be appropriately mounted and inscribed.

Unanimously adopted.

Dr. C. N. Pierce offered an amendment to the constitution, vesting the choice of place of meeting in the executive committee, which lays upon the table for one year.

The Committee on Necrology reported suitable resolutions respecting the death of Dr. Isaiah Forbes, of St. Louis, and of Dr. J. G. Ambler, of New York, which were unanimously adopted.

Dr. A. O. Hunt, from the Section on Dental Education, read a report approving the action of the Association of College Professors with regard to improving the literature of the profession, its text-books, etc. Also their action with reference to equivalent courses in medical colleges, and the diplomas of colleges or high schools.

The report was adopted.

Drs. Geo. L. Field and W. N. Morrison then conducted the President elect to the chair. The retiring President presented him with the battered and broken gavel, and asked for his successor the same kind consideration he himself had received.

Dr. Barrett accepted the office with a few brief but appropriate words. He thanked the Association for the great but unsolicited honor, saying that he would be either more or less than human if he failed to appreciate, tenderly and heartily, the compliment paid him.

The President announced that he would appoint a local committee as soon as the place of meeting should be determined.

The Secretary, Dr. Cushing, Dr. A. W. Harlan, and Dr. E. T. Darby, were appointed a Committee on Publication.

The Association then adjourned *sine die.*

### THE EXCURSION.

At 2 P. M., the members, accompanied by a large number of ladies and invited guests, assembled for the last time at Curtiss Hall, and proceeded to the depot of the Minneapolis and St. Louis R. R., where a special train of seven coaches was waiting to convey them to Lake Minnetonka. Arriving at the lake they boarded the fine steamer, Belle of Minnetonka, and made the tour of the lovely lake, touching at Excelsior, Hotel St. Louis, and other points. The weather was delightful, the sunset gorgeous, and twilight deepened into darkness ere the Lake Park Hotel was reached, where an elegant dinner awaited them.

Numerous toasts were proposed and responded to, Dr. A. W. Spaulding, of Minneapolis, acting as toast-master. The committee of arrangements had done their duty so perfectly that the occasion was one of unmixed pleasure. The next morning, by special invitation, a large party visited the celebrated flouring mills of Minneapolis. Many visited the lovely Falls of Minnehaha, noted in poetry and song. Others spent the day at St. Paul. On Saturday quite a numerous party left for the Yellowstone Park. The last lingerers turned their faces homeward, and the Twenty-fifth Annual Session of the American Dental Association became a thing of the past, long to be pleasantly remembered.

